# Pressure pain threshold and somatosensory abnormalities in different ages and functional conditions of post-stroke elderly

**DOI:** 10.1186/s12877-022-03515-4

**Published:** 2022-10-28

**Authors:** Yong-Hui Zhang, Hao-Ran Xu, Yu-Chen Wang, Gong-Wei Hu, Xiao-Qin Ding, Xiao-Hua Shen, Hui Yang, Ji-Feng Rong, Xue-Qiang Wang

**Affiliations:** 1 Department of Sport Rehabilitation, Shanghai University of Sport, 399 Changhai RD, Shanghai, 200438 China; 2 The Center of Rehabilitation Therapy, The First Rehabilitation Hospital of Shanghai, 349 Hangzhou RD, Shanghai, 200090 China; 3 Department of Rehabilitation Medicine, Shanghai Shangti Orthopaedic Hospital, Shanghai, China

**Keywords:** Age, Elderly, Pain threshold, Stroke, Somatosensory, Hemiplegia

## Abstract

**Background:**

Somatosensory deficits and abnormal pain sensitivity are highly prevalent among stroke survivors, which negatively impacts their quality of life and recovery process. However, the factors for pressure pain threshold (PPT) and somatosensory abnormalities in post-stroke elderly remain unknown. The aim of this study was to explore the effects of age, side and other functional conditions, such as spasticity and motor functions, on PPT and sensory abnormalities among elderly after stroke.

**Methods:**

The cross-sectional study finally included 43 post-stroke elderly aged over 60 and assessed the PPT of 14 bilateral muscles widely located in the whole body by using a digital force gage. Meanwhile, spasticity, motor function, joint pain and activity of daily living (ADL) were evaluated by the Modified Ashworth scale, Fugl-Meyer, and Barthel Index, respectively. All participants were divided into higher-aged and lower-aged groups based on the median age of all of them.

**Results:**

Higher age tended to be associated with higher sensitivity but not significant except for one upper limb muscle, and the affected side showed significantly higher PPTs than the unaffected side in three out of seven muscles (*p* < 0.05). Furthermore, the somatosensory abnormalities in the affected side, particularly hypoalgesia, were more frequent in higher-aged than lower-aged patients in most assessed muscles. Meanwhile, patients with spasticity showed more increment of PPTs in affected muscles around the knee joint than patients without spasticity (*p* < 0.05). Patients with better motor functions, less joint pain and higher ADL performed less bilateral differences of PPTs than other patients in some muscles (*p* < 0.05).

**Conclusions:**

The age and side differences of mechanical pain sensitivity were found among post-stroke elderly. Older patients show higher sensitivity in both sides compared with the younger ones, and the affected side of the elder shows more somatosensory abnormalities, particularly hypoalgesia, than that of the younger ones. Post-stroke elderly in good functional conditions, such as normal muscle tone, better physical function and daily activities, and less joint pain, seems to have more equal pain sensitivity between both sides than those in poor conditions.

**Supplementary Information:**

The online version contains supplementary material available at 10.1186/s12877-022-03515-4.

## Introduction

Stroke is a high-prevalence disease worldwide, which causes around 110 million disability-adjusted life years and 5.5 million deaths in a single year [1]. In 2011, about $33 billion were cost for stroke, which placed a heavy economic burden on the USA government [2]. Neurological deficiencies of stroke primarily include spasticity, motor impairment, cognitive disorder and sensory abnormality. In particular, somatosensory impairment is a common disability after stroke, with a 50 to 80% of prevalence [3]. Statistically, at least one kind of somatosensory deficits is found in around 60% of stroke patients. Somatosensory modalities could be classified into exteroception and proprioception. To elaborate, exteroception is generated by analyzing external skin information whereas proprioception deals with internal movement and position information from muscle tendon and joint [3]. Post-stroke patients with somatosensory deficits might have difficulties in light touch detection, two-point discrimination and temperature sensation [4]. Meanwhile, somatosensory abnormality could result in pain, which was reported in 19–74% of stroke patients [5–8]. Nevertheless, although around 60% of the patients present sensory deficits, sensory retraining is frequently overlooked in the treatment plan during rehabilitation [9]. As one sort of somatosensory modalities, pain perception abnormality has been proven to be related to the development of pain after brain lesion [10]. Thus, the assessment of pain threshold may be significant for clinical staff to treat post-stroke patients with sensorimotor dysfunction or pain.

Many previous studies have researched pain perception in different populations. According to Mücke et al., the increases in thermal and vibration pain perception were strongly associated with aging, which indicates that age is positively associated with pain perception in healthy population [11]. Moreover, no left–right side difference was observed, except for tissue influence of pain perception among healthy subjects [12]. Furthermore, patients with high intensity of neck pain performed sensitive mechanical pain perception, whereas sleep quality and symptom duration were not associated with pain perception [13]. In addition, sex was extensively reported as an influencing factor of pressure pain perception in healthy and stroke subjects, with a higher sensitivity in female [11, 14–16]. Somatosensory modalities, such as pain perception, plays a crucial role in body function and goal-directed action in daily activities of stroke survivors [17]. However, few high-quality research studies have reported the relationship between pain perception and other functions. Although pressure pain has been researched in healthy population and different patients, a few studies have focused on pressure pain threshold (PPT) in post-stroke elderly and its factors.

This study aimed to explore the age and side effects on pressure pain sensitivity in elder stroke survivors through assessing the PPT of large muscles located all over the body in affected and unaffected sides among 43 patients aged over 60. Moreover, this study aimed to evaluate the relationships between pain sensitivity for pressure stimuli and functional conditions, such as spasticity, motor functions and ADL, in elderly after stroke.

## Methods

### Study design

Forty-three participants were involved in this cross-sectional study. Fourteen points in twelve muscles on both sides, including the middle deltoid muscles (MD), biceps brachii muscles (BB), erector spinae muscles (ES) at the second and fourth lumbar vertebra (L2 and L4) levels, rectus femoris muscles (RF), biceps femoris muscles (BF), and medial gastrocnemius muscles (MG), were selected to evaluate PPT. In addition, spasticity, motor function, joint pain, and ADL were evaluated by the Modified Ashworth Scale (MAS), upper limb (FM-UL), lower limb (FM-LL), motor function (FM-MF), and joint pain (FM-JP) subscales of Fugl-Meyer Assessments and Barthel Index, respectively. In addition, other baseline information was collected for further exploration. The study was approved by Human Ethics Committee of the First Rehabilitation Hospital of Shanghai (YK-2020–01-030). All willing participants signed informed consent forms.

### Participants

According to the mean values and standard deviation values from a previous study, G*Power was used to evaluate the sample size, with Mann–Whitney U test, α = 0.05, power = 0.8, allocation ratio N2/N1 = 1. The total sample size was 36 with an actual power of 0.81 [18]. This study recruited post-stroke patients who received similar rehabilitation in the First Rehabilitation Hospital of Shanghai. The inclusion criteria were: (1) aged over 60, (2) stroke onset more than 1 month, (3) diagnosed as hemorrhage or ischemia, and (4) able to keep a prone position for 15 min. The exclusion criteria were: (1) with cognitive disorder that may cause latency or inaccuracy of response to pressure, (2) disobedient behavior during the test, (3) any other diseases which may cause neurological symptoms, (4) have been under any pain relief treatment within a month or having any analgesics or antispastics.

### Outcome assessments

PPT was defined as the turning point when pressure sensation transformed into pain sensation [19]. PPT assessment could be diagnostic and instructive for clinicians facing patients with impaired neural pathway processing and abnormal sensory perception [20, 21]. A Digital Force Gage (Wagner FDX-25, Greenwich, CT) was used to evaluate PPT by only one operator from the beginning to the end of the experiment. The operator applied a gradually increasing force perpendicular to the skin through a 1-cm^2^ rubber tip at a stable speed until the PPT was reached. The apparatus showed a good-to-excellent test–retest and inter-rater reliability based on the previous research (intraclass correlation coefficient, 0.76–0.97) and has been widely applied to the clinical measurement of PPT [13, 22]. The unit of this apparatus was kgf/cm^2^, which ranged from 0 to 14 kgf/cm^2^. In addition, MAS was used to measure the spasticity of flexors or extensors in different joints by moving the patient limb from a position of maximal flexion to maximal extension or moving through opposite direction. Moreover, motor functions of the upper and lower limb, such as active movement of the shoulder and hip, were evaluated by FM-UL and FM-LL, respectively. Afterward, the overall motor function (FM-MF) was calculated by the sum of FM-UL and FM-LL. The FM-JP was applied by recording pain severity during passive joint movement. Finally, the ADL, such as dressing, transfers and stairs, was evaluated by the Barthel Index.

### Study paradigm

Before the PPT test, participants were instructed to lie on a bed in supine position and informed with the procedures of the following PPT test. Five-time practice was available for participants to get familiar with it. Participants were required to give their response as soon as they feel the sensation transforming. After practice, palpation conducted by the operator was required to help mark the test points on the belly of targeted muscles. The muscle belly was selected in this study because muscle tissue showed the least changes of PPT when different kinds of evaluation apparatus and circumstances were applied, which might indicate a better consistency in various clinical practices compared with other tissues (tendon, bone, and nail) [11, 12]. All 14 points were marked by a black pen to eliminate bias caused by evident location inaccuracy. The stimuli were applied on the marked area perpendicular to skin. Pressure stimuli were given in a non-overlapping adjacent area to prevent soft tissue from fatigue and deformation. During formal PPT assessments, the participants were given pressure stimuli to MD, BB, and RF muscles by the operator. Then, they were asked to shift into prone position, and ES (L2), ES (L4), BF, and MG muscles were assessed in turn. For all participants, PPT tests were standardized to start at the unaffected side. Three repetitions were applied for each point and the interval between two repetitions was approximately 30 s.

### Statistical analysis

SPSS statistics (IBM SPSS 22.0, Chicago, IL) was applied to analyze the data. All participants were divided into the lower-aged group and higher-aged group in accordance with the median age (median age = 71). The Mann–Whitney U test and Chi-square test were used to detect any differences for qualitative and quantitative demographics between two groups, respectively. Adjusted liner regression analysis was used to detect the association between age and PPT values and the adjusted factors were sex, body mass index, and stroke type. In comparing PPT values between the unaffected and affected sides, normal transformation and paired-sample T test were performed.

In a previous study [10], pressure pain perception seems to be abnormal when the ratio of bilateral PPT values is over 136% or less than 74%. This study analyzed the ratios of PPT value in the affected side to the value in the unaffected side and defined hyperalgesia when the ratio was less than 74% and hypoalgesia when over 136%, which were considered as positive somatosensory abnormalities. Fisher’s exact test was applied to find the influence of age on the frequencies of positive sign, including hyperalgesia, hypoalgesia, and negative sign since the expected values were less than 5.

Except for the ratio, the absolute difference values of PPT between the affected and unaffected sides were calculated to present the bilateral differences. Subsequently, based on the MAS results of flexors and extensors at different joints to detect the effect of muscle spasticity on PPTs, all participants were divided into two groups, normal group (MAS = 0) and spasticity group (MAS > 0). The Mann–Whitney U test was applied to find the PPT differences between normal and spasticity groups. Finally, a two-tailed spearman test was used to find the correlation between the functions and PPT based on the results from functional scales. Notably, the Chi-Square test was applied to detect any sex imbalance between the normal group and spasticity group because sex has been demonstrated to be a factor for PPT in previous research. Similarly, the correlations between PPT and functions among male and female participants were provided separately.

## Results

Forty-three patients were included in the study and the baseline information of them is demonstrated in Table 1. The median age of all participants was 71 and the median BMI was 23.24 kg/m^2^. No significance of demographic characteristics was found between the lower-aged group and higher-aged group.

**Table 1:** Demographics and characteristics of all post-stroke elderly

	**All participants (*n* = 43)**	**Lower-aged (*n* = 19)**	**Higher-aged (*n* = 24)**	***p***
Sex, male n (%)	27 (62.8%)	14 (73.7%)	13 (54.2%)	0.189
BMI (kg/m^2^)	23.24 (21.48–26.03)	23.89 (22.60–27.34)	23.14 (21.27–24.31)	0.276
Smoking n (%)	21 (48.8%)	12 (63.2%)	9 (37.5%)	0.182
Drinking n (%)	16 (37.2%)	11 (57.9%)	5 (20.8%)	0.409
Days between stroke onset and assessment	324.00 (195.00–537.00)	489.00 (230.00–726.00)	295.5 (127.75–471.50)	0.053
Stroke type				0.326
*Hemorrhage n (%)*	11 (25.6%)	8 (42.1%)	3 (12.5%)	N/A
*Ischemia n (%)*	32 (74.4%)	11 (57.9%)	21 (87.5%)	N/A
Right-side affected n (%)	19 (44.2%)	8 (42.1%)	11 (45.8%)	0.807
MMSE	28.00 (22.00–30.00)	29.00 (21.00–30.00)	26.00 (22.00–30.00)	0.478
Barthel Index	65.00 (45.00–85.00)	65.00 (45.00–85.00)	67.00 (46.25–84.75)	0.893
FM-UL	18.00 (11.00–53.00)	15.00 (6.00–36.00)	20.00 (16.25–53.00)	0.101
FM-LL	20.00 (13.00–27.00)	15.00 (9.00–20.00)	20.50 (12.25–27.50)	0.962
FM-MF	40.00 (25.00–77.00)	21.00 (14.00–30.00)	42.00 (29.25–77.75)	0.365
FM-JP	38.00 (30.00–44.00)	30.00 (18.00–36.00)	38.00 (24.50–44.00)	0.748
FMA-BF	10.00 (8.00–11.00)	7.00 (4.00–10.00)	10.00 (8.00–11.00)	0.776
FMA-SF	20.00 (14.00–24.00)	14.00 (10.00–21.00)	19.50 (12.50–24.00)	0.664
FMA-ROM	37.00 (28.00–44.00)	30.00 (27.00–34.00)	37.50 (24.00–44.00)	0.775

All elderly were divided into the lower-aged and the higher-aged groups based on the median age (71) of them

*BMI* Body mass index, *MMSE* Mini-Mental State Exam, *FM* Fugl-Meyer Assessment, *UL* Upper limb, *LL* Lower limb, *MF* Motor function, *JP* Joint pain, *BF* Balance function, *S* Sensory function, *ROM* RZange of motion of joints

Data were presented as median (Q1-Q3) or as the number of participants (%). *p*-values came from the Mann–Whitney U-test for quantitative data and the Chi-square test for qualitative data

### Age and side differences

**Fig. 1:**
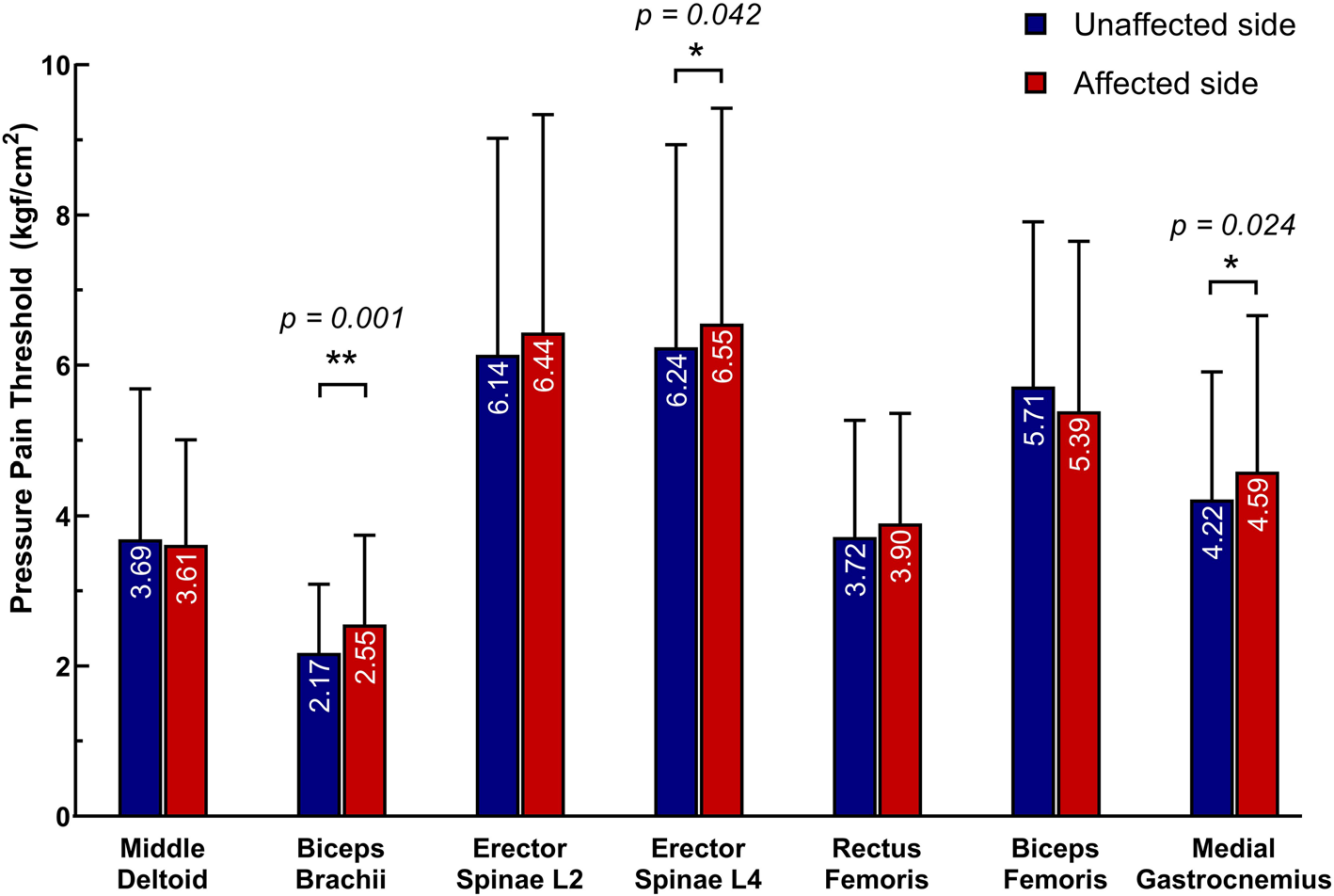
The comparison of PPTs between both sides among all participants. The biceps brachii, erector spinae at the L4 level, and medial gastrocnemius muscles in affected side showed significantly higher PPTs than those in the unaffected side (*p* < 0.05); * meaning *p* < 0.05, ** meaning *p* < 0.01. The data were presented as means with SDs. The numbers in the bar were the mean values of PPTs

**Fig. 2:**
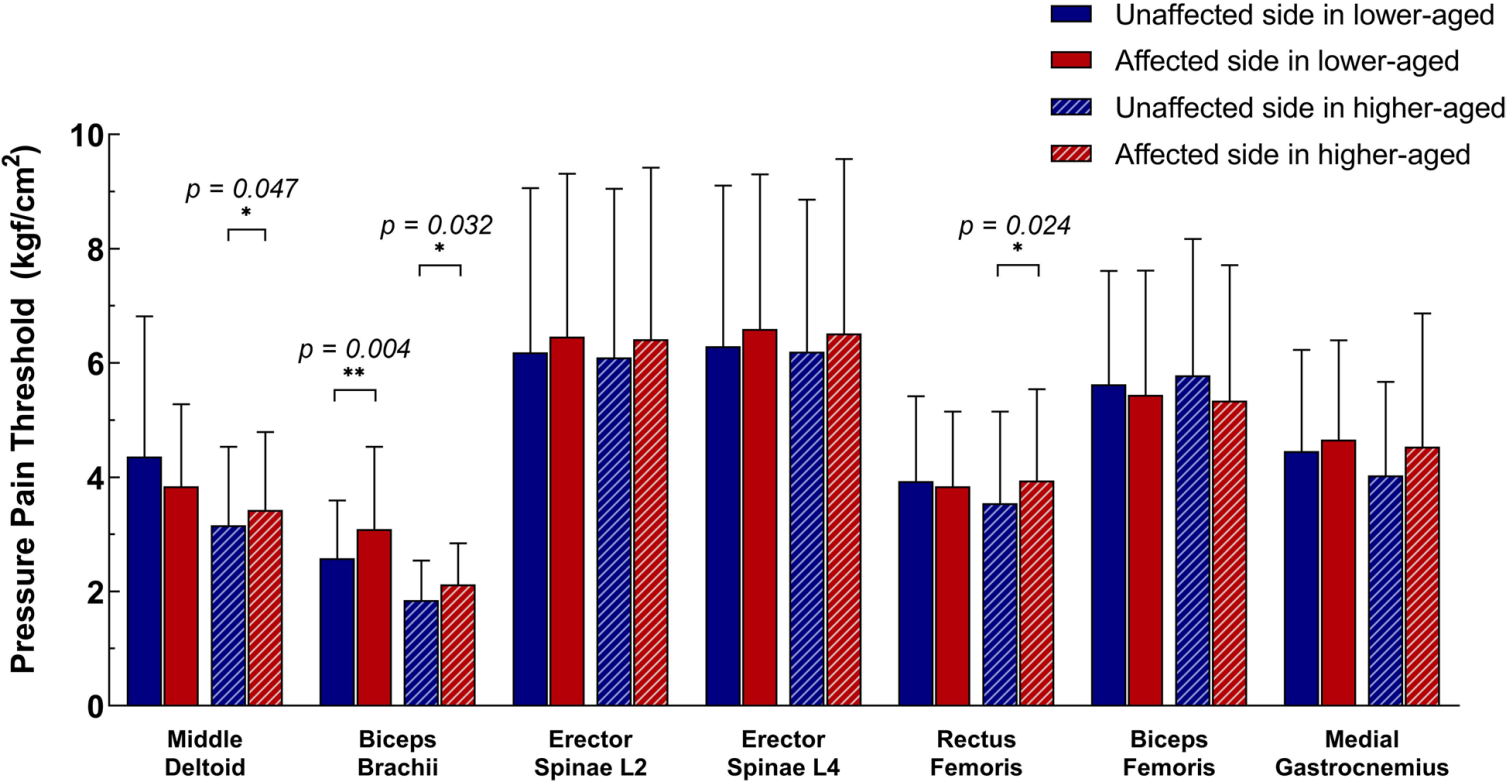
The comparison of PPTs between both sides in the lower-aged and the higher-aged elderly. All elderly were divided into lower-aged and higher-aged groups based on the median age (71) of them. The biceps brachii muscles in both groups showed side differences (*p* < 0.05); The middle deltoid and rectus femoris muscles in the higher-aged groups showed side differences (*p* < 0.05); All side differences were statistically significant increases of PPTs in the affected side compared to those in the unaffected side. * meaning *p* < 0.05, ** meaning *p* < 0.01. The data were presented as means with SDs

The associations between age and PPT of different muscles are provided in Table S1 in supplementary 1 and the comparisons of PPT of measured muscles between both sides are demonstrated in Figs. 1 and 2. Although higher age tended to be associated with lower PPT values regardless of sides, but not significant (*p* > 0.05) in the regressions except for the BB in the unaffected side (*p* = 0.028). In addition, nearly all PPT values in the affected side were higher than those in the unaffected side not only in all participants but also in different age groups. In all participants, side differences of BB, ES (L4), and MG were significant (*p* < 0.05). Furthermore, one out of seven muscles (BB) showed a side difference in the lower-aged group (*p* < 0.05), whereas three out of seven muscles (MD, BB, and RF) showed this difference in the higher-aged group (*p* < 0.05). Almost all significant side differences were found in muscles located in the upper and lower extremities instead of the trunk.

### Somatosensory signs

Different from comparison between two sides among all patients, the results of hyperalgesia and hypoalgesia analyses providing the side comparison at individual level are demonstrated in Table 2. Hypoalgesia was more frequently observed than hyperalgesia among all participants, and it occurred more in the higher-aged group than in the lower-aged group. Based on Fisher’s exact tests, the percentage of hyperalgesia, hypoalgesia and negative signs in MD and RF was significantly different between the lower- and higher-aged groups. In addition, a more frequent occurrence of positive sign was found in muscles located in the upper and lower extremities than in the trunk.

**Table 2:** Positive and negative somatosensory signs for pressure stimuli in lower-aged, higher-aged and all elderly (n, %)

	**Positive Sign**			**Negative Sign**	***p*1**	***p*2**
	**Hyperalgesia**	**Hypoalgesia**	**All**			
***Lower-aged (n* = *19)***						
Middle Deltoid	6 (31.58%)^a^	2 (10.53%)^b^	8 (42.11%)	11 (57.89%)^b^	0.013*	0.091
Biceps Brachii	0 (0)	5 (26.32%)	5 (26.32%)	14 (73.68%)	0.642	0.743
Erector Spinae L2	0 (0)	1 (5.26%)	1 (5.26%)	18 (94.74%)	1.000	1.000
Erector Spinae L4	0 (0)	2 (10.53%)	2 (10.53%)	17 (89.47%)	0.492	1.000
Rectus Femoris	1 (5.26%)^a^	0 (0)^b^	1 (5.26%)	18 (94.74%)	0.027*	0.112
Biceps Femoris	1 (5.26%)	0 (0)	1 (5.26%)	18 (94.74%)	0.267	0.112
M Gastrocnemius	1 (5.26%)	0 (0)	1 (5.26%)	18 (94.74%)	0.148	0.205
***Higher-aged (n* = *24)***						
Middle Deltoid	0 (0)	4 (16.67%)	4 (16.67%)	20 (83.33%)	N/A	N/A
Biceps Brachii	2 (8.33%)	6 (25.00%)	8 (33.33%)	16 (66.67%)	N/A	N/A
Erector Spinae L2	0 (0)	2 (8.33%)	2 (8.33%)	22 (91.67%)	N/A	N/A
Erector Spinae L4	2 (8.33%)	1 (4.17%)	3 (12.50%)	21 (87.50%)	N/A	N/A
Rectus Femoris	0 (0)	6 (25.00%)	6 (25.00%)	18 (75.00%)	N/A	N/A
Biceps Femoris	3 (12.5%)	3 (12.5%)	6 (25.00%)	18 (75.00%)	N/A	N/A
M Gastrocnemius	1 (4.17%)	4 (16.67%)	5 (20.83%)	19 (79.17%)	N/A	N/A
***All Participants (n* = *43)***						
Middle Deltoid	6 (13.95)	6 (13.95%)	12 (27.91%)	31 (72.09%)	N/A	N/A
Biceps Brachii	2 (4.65%)	11 (25.58%)	13 (30.23%)	30 (69.77%)	N/A	N/A
Erector Spinae L2	0 (0)	3 (6.98%)	3 (6.98%)	40 (93.02%)	N/A	N/A
Erector Spinae L4	2 (4.65%)	3 (6.98%)	5 (11.63%)	38 (88.37%)	N/A	N/A
Rectus Femoris	1 (2.33%)	6 (13.95%)	7 (16.28%)	36 (83.72%)	N/A	N/A
Biceps Femoris	4 (9.30%)	3 (6.98%)	7 (16.28%)	36 (83.72%)	N/A	N/A
M Gastrocnemius	2 (4.65%)	4 (9.30%)	6 (13.95%)	37 (86.05%)	N/A	N/A

All elderly were divided into lower-aged and higher-aged groups based on the median age (71) of them. Data were presented as numbers of subjects (% of participants). Positive signs were determined by the ratios of PPTs (affected/unaffected). Hypoalgesia: ratio > 136%; Hyperalgesia: ratio < 74%; *p*1-values came from the Fisher’s exact test to detect the age effect on the frequencies of hyperalgesia, hypoalgesia and negative sign. ‘a’ and ‘b’ represented the subgroups that showed significant differences in this 2 × 3 Fisher’s exact test. *p*2-values came from the Fisher’s exact test to detect the age effect on the frequencies of positive sign and negative sign, which was no significant difference (*p* > 0.05)

### Spasticity difference

Outcome with significant differences between the normal and spasticity groups based on MAS is reported in Table 3 and all results are provided in Table S2 in supplementary 1. The outcome of Chi-square test showed no difference of sex composition among the groups. When classifying by the muscle tone of elbow extensors, the bilateral difference of ES (L2) muscles in the spasticity group was higher than that in the normal group. Based on the muscle tone of knee flexors, the bilateral difference of ES (L4) muscles and the ratios of RF showed significant differences between the normal and spasticity groups. Based on knee extensors, the ratio in RF muscle in the spasticity group was significantly higher than that in the normal group.

**Table 3:** All significant differences between post-stroke elderly with and without spasticity

	***p*1**	**Normal Group**	**Spasticity Group**	***p*2**
**Elbow Extension**	0.495	***n* = 16 (M = 9, 56.25%)**	***n* = 27 (M = 18, 66.67%)**	
Erector Spinae L2 (BD)		0.465 (0.183–0.770)	0.740 (0.417–1.207)	0.034*
**Knee Flexion**	0.252	***n* = 22 (M = 12, 54.55%)**	***n* = 21 (M = 15, 71.43%)**	
Erector Spinae L4 (BD)		0.552 (0.287–0.667)	0.953 (0.367–1.600)	0.026*
Rectus Femoris (%)		95.26 (85.73–108.42)	113.78 (97.13–124.69)	0.006**
**Knee Extension**	0.555	***n* = 19 (M = 11, 57.89%)**	***n* = 24 (M = 16, 66.67%)**	
Rectus Femoris (%)		93.5 (82.89–103.31)	112.26 (97.01–125.77)	0.003**

All participants were divided into normal and spasticity group according to the result of Modified Ashworth assessment. Those who were assessed as ‘0’ in certain muscle were distributed to normal group and others were distributed to spasticity group. BD, bilateral difference of PPT values (affected–unaffected); %, ratio of PPT values (affected/unaffected). *p*1-values came from the Chi-square test to show there is no significant difference of sex composition between groups (*p* > 0.05). *p*2*-*values came from the Mann–Whitney U-test

### Functions and PPT

**Fig. 3:**
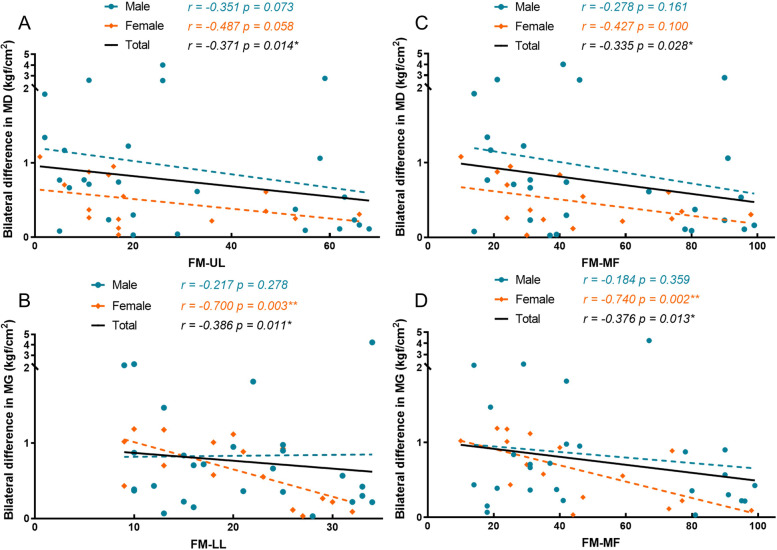
The significant correlations between bilateral difference and motor function. MD, middle deltoid muscle; MG, medial gastrocnemius muscle; FM, Fugl-Meyer Assessment; UL, upper limb; LL, lower limb; MF, motor function. *r* and *p* values came from the spearman tests. Among all elderly, there were significant correlations between the bilateral difference of PPT in MD and FM-UL (**A**), MG and FM-LL (**B**), MD and FM-MF (**C**), and MG and FM-MF (**D**) (*p* < 0.05)

**Fig. 4:**
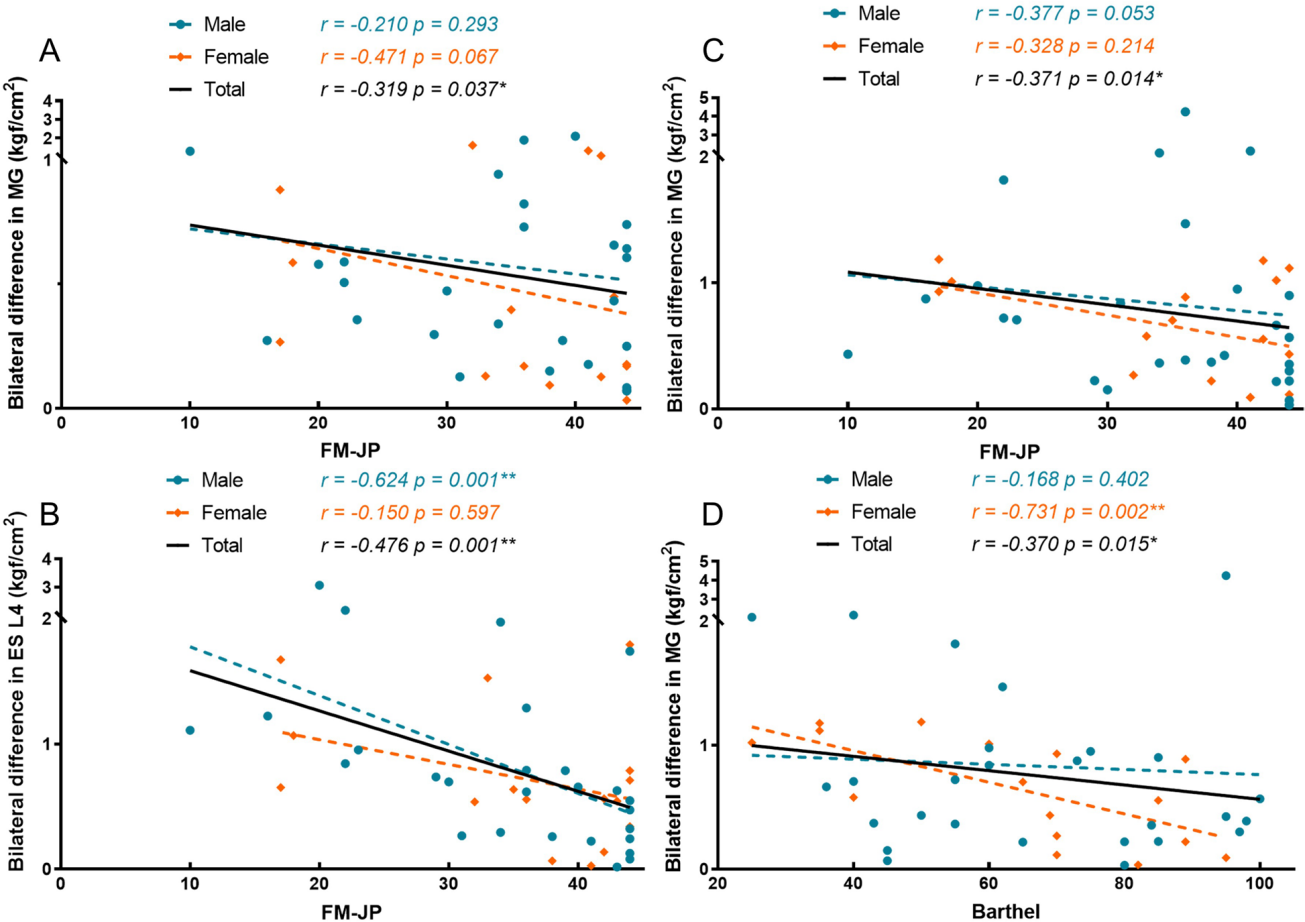
The significant correlations between bilateral difference and functional conditions. BB, biceps brachii muscle; ES L4, erector spinae muscle at L4 levels; MG, medial gastrocnemius muscle; FM, Fugl-Meyer Assessment; JP, joint pain. *r* and *p* values came from the spearman tests. Among all elderly, there were significant correlations between the bilateral difference of PPT in BB (**A**), ES L4 (**B**) and MG (**C**) and FM-JP, and between the bilateral difference of PPT in MG and Barthel Index (**D**) (*p* < 0.05)

Significant correlations (*p* < 0.05) between bilateral difference and functional scales (Fugl-Meyer and Barthel Index) are illustrated in Figs. 3 and 4. All results from spearman tests and significant correlations between ratio and functional scales are provided in Table S3 and Figures S1–5, respectively (supplementary 1). The bilateral difference of MD showed a weak correlation with FM-UL (*r* =  − 0.371, *p* = 0.014) and FM-MF (*r* =  − 0.335, *p* = 0.028). In addition, bilateral difference of MG weakly correlated with FM-LL (*r* =  − 0.386, *p* = 0.011), FM-MF (*r* =  − 0.376, *p* = 0.013), FM-JP (*r* =  − 0.371, *p* = 0.014), and Barthel Index (*r* =  − 0.370, *p* = 0.015). Furthermore, a weak relationship was observed between the bilateral difference of BB and FM-JP (*r* =  − 0.319, *p* = 0.037), and a moderate correlation was found between that of ES (L4) and FM-JP (*r* =  − 0.476, *p* = 0.001).

## Discussions

The study evaluated pain sensitivity for pressure stimuli among 43 stroke survivors over 60 years old. The results showed that higher age might be more sensitive to pressure pain in both sides despite no significances except for one upper limb muscle, whereas older elderly was at higher risk of sensory loss and hypoalgesia in the affected side when compared with the unaffected side. Furthermore, participants with normal muscle tone, less joint pain, and high level of motor function and ADL performed more symmetrical pain perception between the affected and unaffected sides.

### Effects of side and age

Nearly all PPT values in affected muscles were higher than those in unaffected muscles, which might indicate an extensive pressure pain sensory loss on the hemiplegic side in patients with stroke. Some previous studies have revealed that somatosensory performance, including tactile, electrical and pressure sensation, in the hemiplegic side decreased compared with the unaffected side [23, 24], which is consistent with our findings. By contrast, a previous study has indicated that no significant difference in PPT was found between the affected side and unaffected side, which seemed to be conflicting with the outcome in this study [16]. This difference could be interpreted by the distinct tested locations because three points on trapezius were measured in their study.

Participants with higher age tended to be more sensitive to pressure pain either in the affected or unaffected sides. Although these associations were not significant except for one upper limb, unaffected BB, this finding is supported by a previous study by Lin C-H et al. They measured the PPT of muscle tendon and bony prominences and reported that higher-aged post-stroke patients had a higher sensitivity to pressure pain but not to heat pain in both sides than lower-aged patients, which indicated the existence of age effect on pain threshold. This effect varied from different characteristics of pain stimuli [16]. Similarly, Lautenbacher et al. reported the age differences of pressure pain sensitivity in upper extremities among healthy population [25]. That is, older people showed significantly lower thresholds compared with the younger ones. While the former focused on the pain threshold at muscles tendon and bony prominences and the latter reported pain perception in the upper limbs. This study explored the PPT on muscle bellies located all over the body. Considering that pain thresholds for pressure stimuli show strong characteristics of tissue specificity and regional differences, our results provided new evidence that age effects have a wide and multi-dimensional influence for pain threshold on various kinds of tissue and regions in post-stroke elderly [11].

Furthermore, lower- and higher-aged post-stroke elderly showed a similar trend with all patients, which is pain threshold had a tendency to increase in the affected side compared with that in the unaffected side. In particular, older old patients after stroke had more muscles that showed significant difference between the two sides than younger old. This finding indicated that higher-aged stroke survivors were more likely to have sensory loss on their affected side. Similarly, hypoalgesia in the affected side was more frequently observed in higher-aged than in lower-aged post-stroke patients, which indicated a consistent trend. This age differences could be explained on the basis of the activity level. Carey et al. stated that the daily activities of stroke survivors with sensory loss in the affected side were significantly reduced compared with those without sensory loss [4]. Meanwhile, activities could induce the emission of neurotrophic factors, involving brain-derived neurotrophic factor (BDNF) and nerve growth factor (NGF), which could promote motor, cognitive and sensory retention in post-stroke patients [26]. Accordingly, long-term dysfunction could lead to maladaptive plasticity of the brain [27]. In this study, less activity in older patients might indicate less neurotrophic factors working for sensory retention, thereby leading to more sensory loss in the affected side.

In addition, the proportion of hyperalgesia in MD in lower-aged patients was higher than that of hypoalgesia, although hyperalgesia in most muscles was relatively less frequent than hypoalgesia. This difference in tendency might be due to the high prevalence of post-stroke shoulder pain. Large amounts of previous studies reported that post-stroke shoulder pain is a common complication with shoulder tissue injury among post-stroke patients, with a 40% prevalence [28, 29]. From the aspect of molecular biology, Ji et al. pointed out that inflammatory mediators, including prostaglandin E2 and NGF, will release when body tissue was damaged, which could change perceived sensitivity and excitability [30]. Referred pain could also have an influence on pain perception, although the tissue was not injured [31]. Post-stroke shoulder pain could influence an extensive region, thereby causing hyperalgesia. Consequently, although the most predominant pain location of hemiplegic shoulder pain in patients was estimated at the supraspinatus (34%), infraspinatus (50%), teres minor (12%), and upper trapezius muscles (20%), MD is still at high risk of hypersensitivity to pain stimuli [32]. However, the reason why hyperalgesia occurred more in lower-aged but not in higher-aged patients remains unclear. Other factors, including depression and cognition dysfunction, should not be missed because they could also have an indirectly impact on somatosensory processing in patients with post-stroke shoulder pain [33, 34].

With regard to muscle locations, trunk muscles were less likely to have sensory abnormality compared with extremity muscles. Based on a study tracing the related pain of patients one year after stroke, the lower extremity was one of the common pain-stricken regions among all post-stroke survivors [35]. Moreover, Fischer et al. concluded that the lumbar paraspinals and gluteus medius muscle of healthy people located in the lower trunk performed the highest PPT, which was consistent with this study [15]. This inherent characteristic might explain the lower variability in trunk muscles when sensory input is interrupted in stroke survivors.

### Effect of spasticity

This study found that post-stroke patients with spasticity of knee flexors and extensors showed significantly higher ratio of PPT than patients without spasticity, which indicated a relative hyposensitivity of pressure pain in affected RF muscles. This finding indicates that spasticity may be associated with sensory loss, particularly in the lower limbs. Some previous studies have reported the similar phenomenon. Peter et al. stated that patients with hemi-hypesthesia were at significantly high risk of potential upper and lower limb spasticity in the future [36]. Similarly, post-stroke spasticity was more commonly observed in patients with sensory deficits for light touch than those without [37]. Of interest, only one muscle regarding knee joint shows this association between pain sensory and spasticity in this study. The possible explanation is that this study focused on pain perception for pressure stimuli, whereas the previous study researched different sensory functions. Furthermore, some studies pointed out that spasticity was positively associated with central post-stroke pain [38], which seems to be conflicted with the finding in this study. Actually, the association between spasticity and central post-stoke pain could be due to central sensitization. In other words, spasticity pain can be regarded as nociceptive pain to protect us against tissue damage at the beginning. However, long-lasting spasticity pain can trigger maladaptive plasticity within the nociceptive system, thereby leading to central sensitization and finally neuropathic pain [39]. In this study, sensory loss for mechanical pain stimuli is not equal to the post-stroke pain caused by central sensitization, although both of them are demonstrated to be associated with spasticity. These two conditions of muscle might exist during two different periods post-stroke or in different muscles in different body parts, which need more studies in the future.

### Correlation between PPT and function

Multiple correlations were found between PPT and functional condition. To be specific, patients with good function are associated with symmetrical pain perception between both sides. Previously, the severity of sensory deficits was proven to be a prognostic factor to predict post-stroke recovery through a large sample regression model [40, 41]. Other studies have supported that somatosensory input is crucial for stroke patients to achieve better motor performance. In other words, stroke patients with somatosensory absence are more likely to suffer from more severe impairments [42, 43]. Based on the theory of neuroplasticity, adequate sensory input could raise the excitability of coactive neurons, and the interconnection of neurons may lead to positive changes in dendritic spines and axons at synapse, which induces brain plasticity, thereby leading to regain. Unignorably, inappropriate sensory gains can trigger unsatisfactory manifestations on motor function [44]. To elaborate, the processing of extracting precise task-relevant sensory information is an indispensable element of motor relearning for post-stroke patients because an optimal strategy for motor task relies on the accurate sensory feedback to a great extent [45]. Therefore, with the recovery of pain sensitivity in the affected side, the abolish circuits might be activated to carry out motor recovery and correct sensory input could improve motor learning, which contribute to better motor outcome.

A more symmetrical pain perception in both sides was found in stroke survivors with less joint pain. Considering that the FM-JP detects joint pain during movement, spasticity characterized by velocity-dependent might primarily account for the induction of pain. Thus, patients with severe spasticity seems to report more joint pain. As mentioned previously, this study reported that patients with spasticity suffered from more sensory loss in the affected side and larger bilateral difference, which is consistent with this finding. Furthermore, the recovery of mechanical pain perception in affected side was associated with the improvement of daily activity. Similarly, previous research indicated that proprioception and tactile sensation deficits were weakly-to-moderately related to independent ADL and recovery of ADL in post-stroke patients, and the combination of two sensations was more strongly associated with daily activity [40, 46]. Meanwhile, sensory loss is an important risk factor for poor daily activity by multiple regression analyses [46]. This study provided new evidence regarding the relationship between ADL and the recovery of mechanical pain sensitivity. Of interest, the significant relationship occurred at MG muscle alone. One speculation is the important role of MG during daily activity, such as transfer, mobility and stairs. Thus, the recovery of sensory input in MG muscle could more directly improve the daily activity of stroke survivors compared with other muscles.

### Limitations

There are some limitations in this study. Firstly, the sample size was small, even though it satisfied the sample size calculation. More participants may provide more information about mechanical pain threshold in difference age ranges in post-stroke elderly and increase the reliability of the results. Meanwhile, the study did not consider the potential influence of stroke type or stroke volume size or location on pressure pain sensitivity, which should be further researched through subgroup analysis or other statistical methods with larger sample size. Thirdly, the study reported the correlation between pain sensitivity and motor function, joint pain condition, and basic ADL, whereas the correlation with more other functional conditions, such as instrumental ADL, mental health, and quality of life were not provided. In future research study, more relationship between pain sensitivity and other functional condition in post-stroke elderly should be explore, and regression analysis could be performed to provide more information.

### Clinical importance

The findings in this study could provide more information about somatosensory performance in post-stroke elderly for clinical staff, which emphasize that older patients are at high risk of hyposensitivity or hypoalgesia in their affected side. Sensory training should be integrated into the rehabilitation prescription for post-stroke elderly, which may be beneficial to preventing potential central or peripheral sensitization, and improving motor function and ADL simultaneously.

## Conclusions

This study explored the age effect of pain sensitivity in post-stroke elderly, which indicated that higher age might be more sensitive to pressure pain both in the affected and unaffected sides but not significant except for one upper limb muscle. Moreover, hyposensitivity and even hypoalgesia were more frequently located at some affected muscles in older elderly than in younger elderly after stroke. Furthermore, post-stroke spasticity seemed to be related with sensory loss in the affected side at knee joint. Elderly after stroke with better motor function, less joint pain during movements, and higher level of ADL showed less sensory loss during pain perception in affected muscles and a more symmetrical pain perception between both sides. In the future, studies involving a large sample size and evaluating more functional conditions in healthy and post-stroke elderly are required.

## Supplementary Information

**Additional file 1.** Associations between age and PPTs in the affected and unaffected sides in **Table S1**. All comparisons of bilateral difference and ratios of PPTs between patients with normal muscles tone and those with spasticity are demonstrated in **Table S2**. The associations between PPTs and functional conditions are shown in **Table S3**. The associations between the subscales of Fugl-Meyer Assessment and ratio of PPTs were illustrated in **Figures S1**, **S2**, **S3**, **S4**, and **S5**.

## Data Availability

The datasets used and/or analyzed during the current study are available from the corresponding author on reasonable request.

## Abbreviations

PPT: Pressure pain threshold
ADL: Activities of daily living
MD: Middle deltoid muscle;
BB: Biceps brachii muscle
ES: Erector spinae muscles
L2: Erector spinae muscle at the second lumbar vertebra level
L4: Erector spinae muscle at the fourth lumbar vertebra level
RF: Rectus femoris muscle
BF: Biceps femoris muscle
MG: Medial gastrocnemius muscle
FM-UL: Upper limb motor function subscale of Fugl-Meyer assessment
FM-LL: Lower limb motor function subscale of Fugl-Meyer assessment
FM-MF: Motor function subscale of Fugl-Meyer assessment
FM-JP: Joint pain subscale of Fugl-Meyer assessment
MAS: Modified ashworth scale; NGF: nerve growth factor
