# Supplementary material for: Pressure pain threshold and somatosensory abnormalities in different ages and functional conditions of post-stroke elderly
Source: BMC Geriatr. 2022 Oct 28;22:830. doi: 10.1186/s12877-022-03515-4 (PMC9617357; doi:10.1186/s12877-022-03515-4)
Supplement: Supplementary file 1 — Additional file 1. Associations between age and PPTs in the affected and unaffected sides in Table S1. All comparisons of bilateral difference and ratios of PPTs between patients with normal muscles tone and those with spasticity are demonstrated in Table S2. The associations between PPTs and functional conditions are shown in Table S3. The associations between the subscales of Fugl-Meyer Assessment and ratio of PPTs were illustrated in Figures S1, S2, S3, S4, and S5. [file 12877_2022_3515_MOESM1_ESM.docx]

**Table S1 Associations between age and PPTs in the affected and unaffected sides**

|  | Affected side | | Unaffected side | |
| --- | --- | --- | --- | --- |
|  | Coefficients of age | *p* values | Coefficients of age | *p* values |
| Middle Deltoid | -0.045 | 0.189 | -0.045 | 0.367 |
| Biceps Brachii | -0.053 | 0.088 | -0.050 | 0.028 |
| Erector Spinae L2 | -0.045 | 0.574 | -0.056 | 0.463 |
| Erector Spinae L4 | -0.048 | 0.546 | -0.045 | 0.534 |
| Rectus Femoris | -0.005 | 0.897 | -0.035 | 0.365 |
| Biceps Femoris | -0.026 | 0.671 | 0.003 | 0.958 |
| M Gastrocnemius | -0.049 | 0.411 | -0.050 | 0.309 |

Notes: coefficients and p values were from adjusted liner regression analysis; adjusted factors were sex, body mass index, and stroke type.

**Table S2 Comparisons of bilateral difference and ratios of PPTs between patients with normal muscles tone and those with spasticity**

|  | ***p* values** | | | | | | | |
| --- | --- | --- | --- | --- | --- | --- | --- | --- |
|  | **Elbow Flexor** | **Elbow Extensor** | **Wrist Flexor** | **Wrist Extensor** | **Knee Flexor** | **Knee Extensor** | **Ankle Flexor** | **Ankle Extensor** |
| **Bilateral Difference** | | | | | | | | |
| Middle Deltoid | 0.28 | 0.97 | 0.196 | 0.816 | 0.725 | 0.854 | 0.931 | 0.95 |
| Biceps Brachii | 0.646 | 0.07 | 0.606 | 0.261 | 0.17 | 0.942 | 0.065 | 0.734 |
| Erector Spinae (L2) | 0.637 | 0.92 | 0.706 | 0.493 | 0.512 | 0.75 | 0.313 | 0.782 |
| Erector Spinae (L4) | 0.398 | 0.034 | 0.269 | 0.06 | 0.409 | 0.392 | 0.136 | 0.725 |
| Rectus Femoris | 0.068 | 0.085 | 0.163 | 0.061 | 0.026 | 0.126 | 0.214 | 0.309 |
| Biceps Femoris | 0.502 | 0.841 | 1 | 0.751 | 0.174 | 0.328 | 0.588 | 0.228 |
| M Gastrocnemius | 0.7 | 0.407 | 0.697 | 0.714 | 0.68 | 0.912 | 0.417 | 0.87 |
| **Ratio** | | | | | | | | |
| Middle Deltoid | 0.518 | 0.421 | 0.98 | 0.696 | 0.697 | 0.293 | 0.902 | 0.315 |
| Biceps Brachii | 0.619 | 0.183 | 1 | 0.448 | 0.099 | 0.493 | 0.237 | 0.563 |
| Erector Spinae (L2) | 0.188 | 0.159 | 0.725 | 0.261 | 0.006 | 0.003 | 0.089 | 0.06 |
| Erector Spinae (L4) | 0.804 | 0.688 | 0.98 | 0.98 | 0.576 | 0.98 | 0.863 | 0.88 |
| Rectus Femoris | 0.785 | 0.482 | 0.9 | 0.625 | 0.308 | 0.591 | 0.98 | 0.96 |
| Biceps Femoris | 0.785 | 0.291 | 0.763 | 0.525 | 0.593 | 0.807 | 0.825 | 0.353 |
| M Gastrocnemius | 0.691 | 0.763 | 0.669 | 0.642 | 0.285 | 0.883 | 0.571 | 0.782 |

Notes: p values from Mann-Whitney U-test to compare the differences between patients with normal muscles tone and spasticity of the specific joint muscles.

**Table S3 The associations between PPTs and functional conditions**

|  |  | **MD** | **BB** | **RF** | **ES (L2)** | **ES (L4)** | **BF** | **MG** |
| --- | --- | --- | --- | --- | --- | --- | --- | --- |
| **Bilateral Difference** | | | | | | | | |
| Barthel | *r* | 0.06 | 0.074 | -0.13 | 0.104 | -0.193 | -0.031 | -0.37 |
|  | *p* | 0.703 | 0.639 | 0.406 | 0.507 | 0.214 | 0.842 | 0.015 |
| FM-UL | *r* | -0.371 | -0.22 | -0.108 | -0.204 | -0.285 | 0.082 | -0.295 |
|  | *p* | 0.014 | 0.156 | 0.49 | 0.19 | 0.064 | 0.601 | 0.055 |
| FM-LL | *r* | -0.157 | -0.016 | 0.186 | -0.008 | -0.178 | -0.142 | -0.386 |
|  | *p* | 0.316 | 0.921 | 0.231 | 0.959 | 0.253 | 0.363 | 0.011 |
| FM-MF | *r* | -0.335 | -0.172 | 0.002 | -0.132 | -0.273 | -0.021 | -0.376 |
|  | *p* | 0.028 | 0.269 | 0.989 | 0.4 | 0.077 | 0.895 | 0.013 |
| FM-JP | *r* | -0.055 | -0.319 | 0.038 | -0.222 | -0.476 | -0.063 | -0.371 |
|  | *p* | 0.727 | 0.037 | 0.811 | 0.153 | 0.001 | 0.688 | 0.014 |
| **Ratio** | | | | | | | | |
| Barthel | *r* | -0.154 | 0.122 | -0.037 | -0.112 | -0.281 | 0.039 | -0.433 |
|  | *p* | 0.324 | 0.437 | 0.813 | 0.473 | 0.068 | 0.803 | 0.004 |
| FM-UL | *r* | -0.344 | -0.071 | -0.118 | -0.299 | -0.407 | -0.117 | -0.304 |
|  | *p* | 0.024 | 0.651 | 0.452 | 0.052 | 0.007 | 0.457 | 0.048 |
| FM-LL | *r* | -0.034 | 0.021 | -0.007 | 0.058 | 0.003 | 0.04 | -0.188 |
|  | *p* | 0.831 | 0.893 | 0.963 | 0.713 | 0.985 | 0.797 | 0.228 |
| FM-MF | *r* | -0.238 | -0.047 | -0.098 | -0.176 | -0.283 | -0.078 | -0.298 |
|  | *p* | 0.124 | 0.763 | 0.533 | 0.26 | 0.066 | 0.621 | 0.052 |
| FM-JP | *r* | -0.181 | -0.24 | -0.088 | -0.053 | -0.191 | -0.147 | -0.314 |
|  | *p* | 0.246 | 0.122 | 0.575 | 0.735 | 0.221 | 0.348 | 0.041 |

Notes: MD, middle deltoid muscle; BB, biceps brachii muscle; ES (L2) and (L4), erector spinae muscle at L2 and L4 levels; RF, rectus femoris muscle; BF, biceps femoris muscle; MG, medial gastrocnemius muscle; FM, Fugl-Meyer Assessment; UL, motor function of upper limb; LL, motor function of lower limb; MF, motor function; JP, joint pain. r and p values came from spearman correlation analyses. Bilateral difference is the absolute PPT value of (affected—unaffected). Ratio represented the ratio of PPT values in affected/unaffected sides.


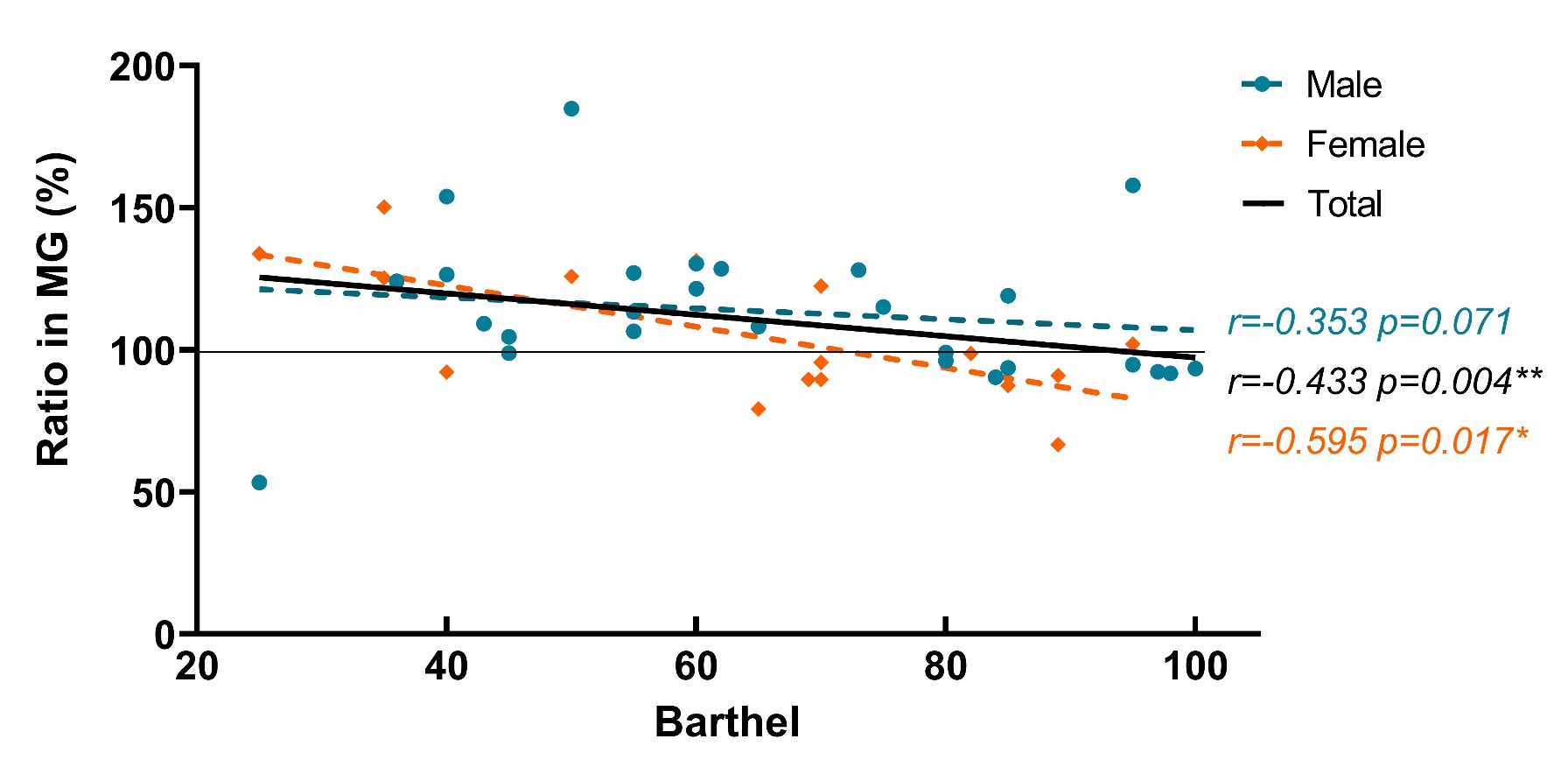


Figure S1 The association between Barthel Index and PPT ratio in medial gastrocnemius muscle among male, female and total post-stroke elderly, respectively


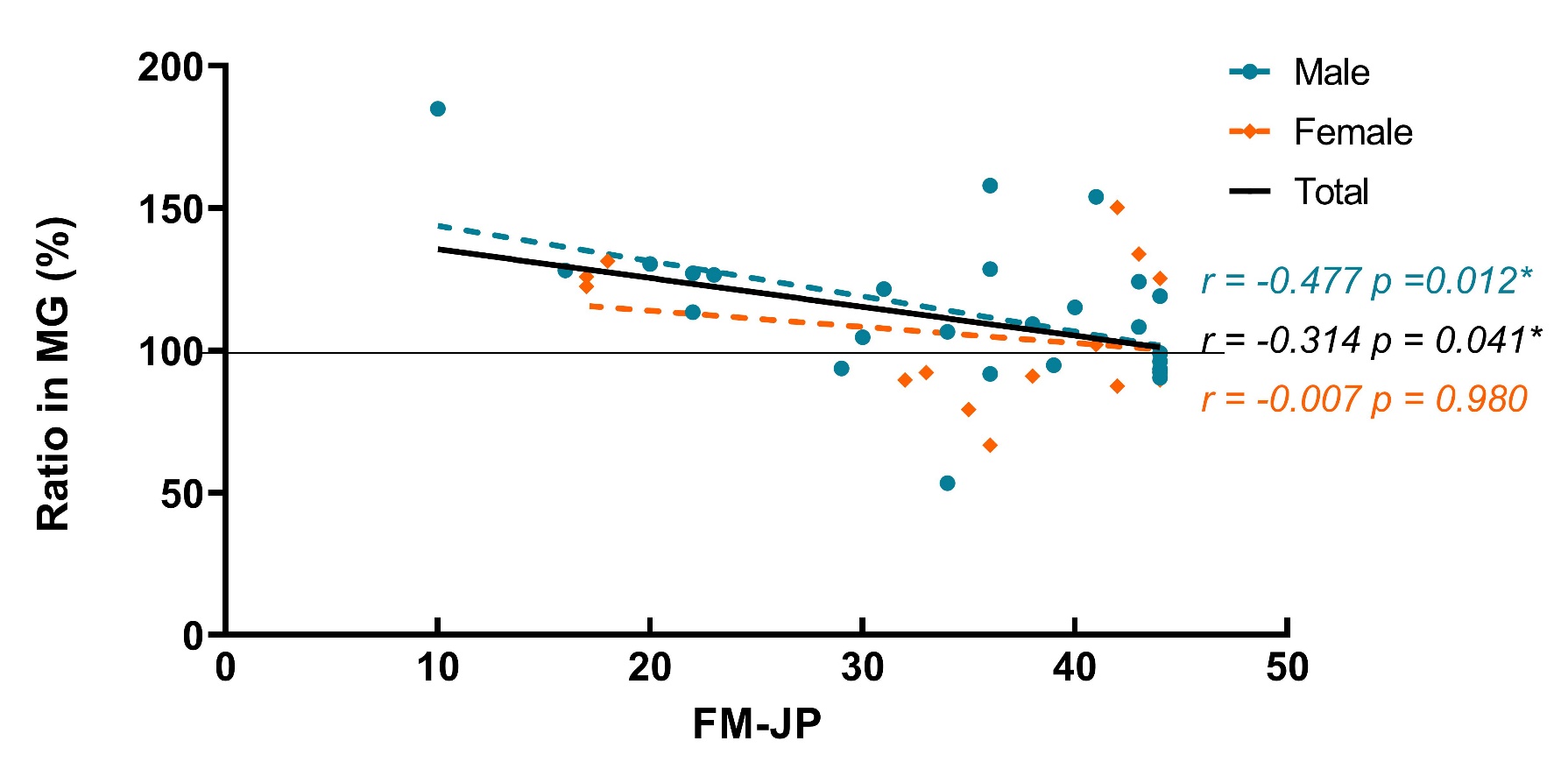


Figure S2 The association between joint pain in Fugl-Meyer Assessment and PPT ratio in medial gastrocnemius muscle among male, female and total post-stroke elderly, respectively


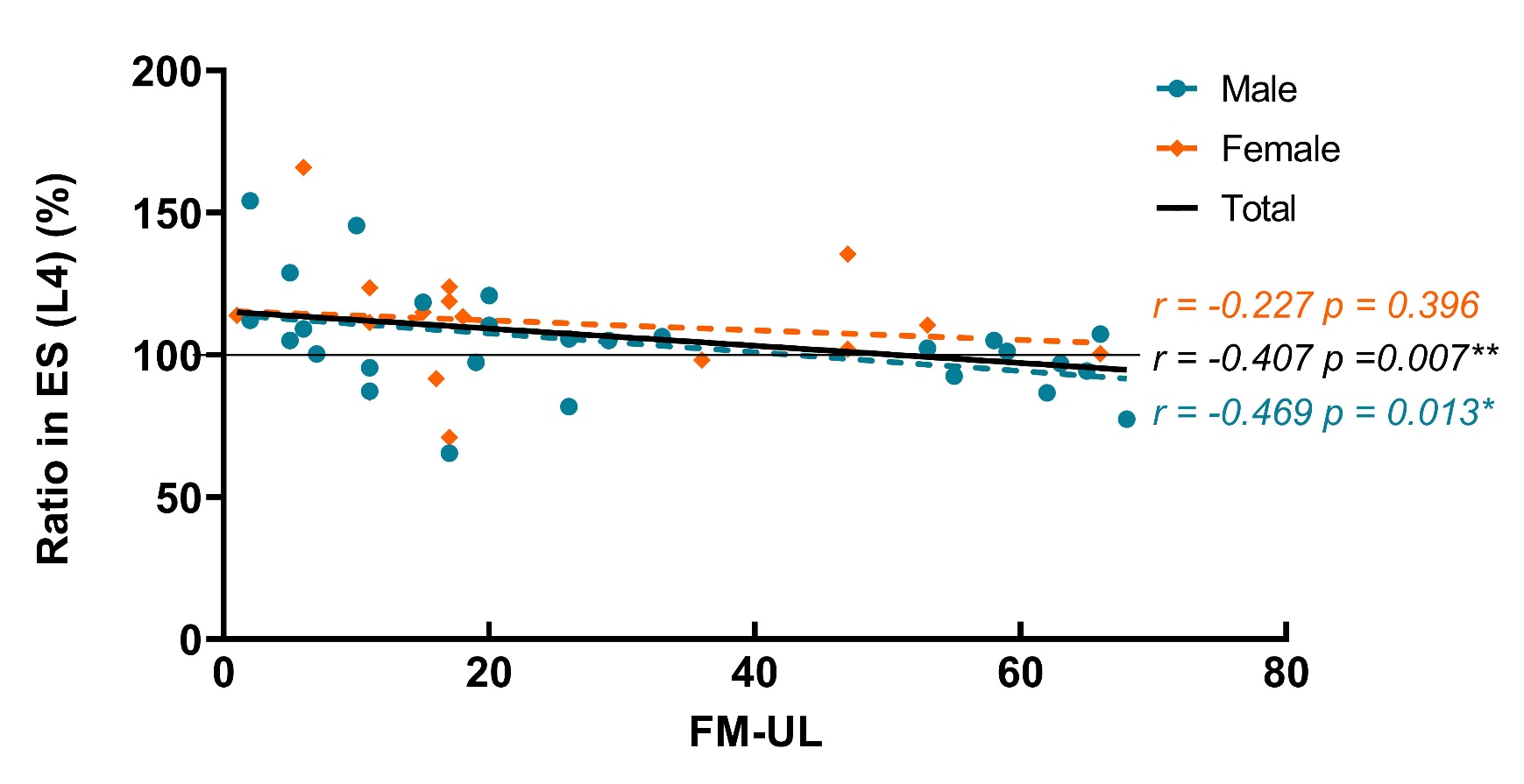


Figure S3 The association between motor function of upper limb in Fugl-Meyer Assessment and PPT ratio in erector spinae muscle at L4 level among male, female and total post-stroke elderly, respectively


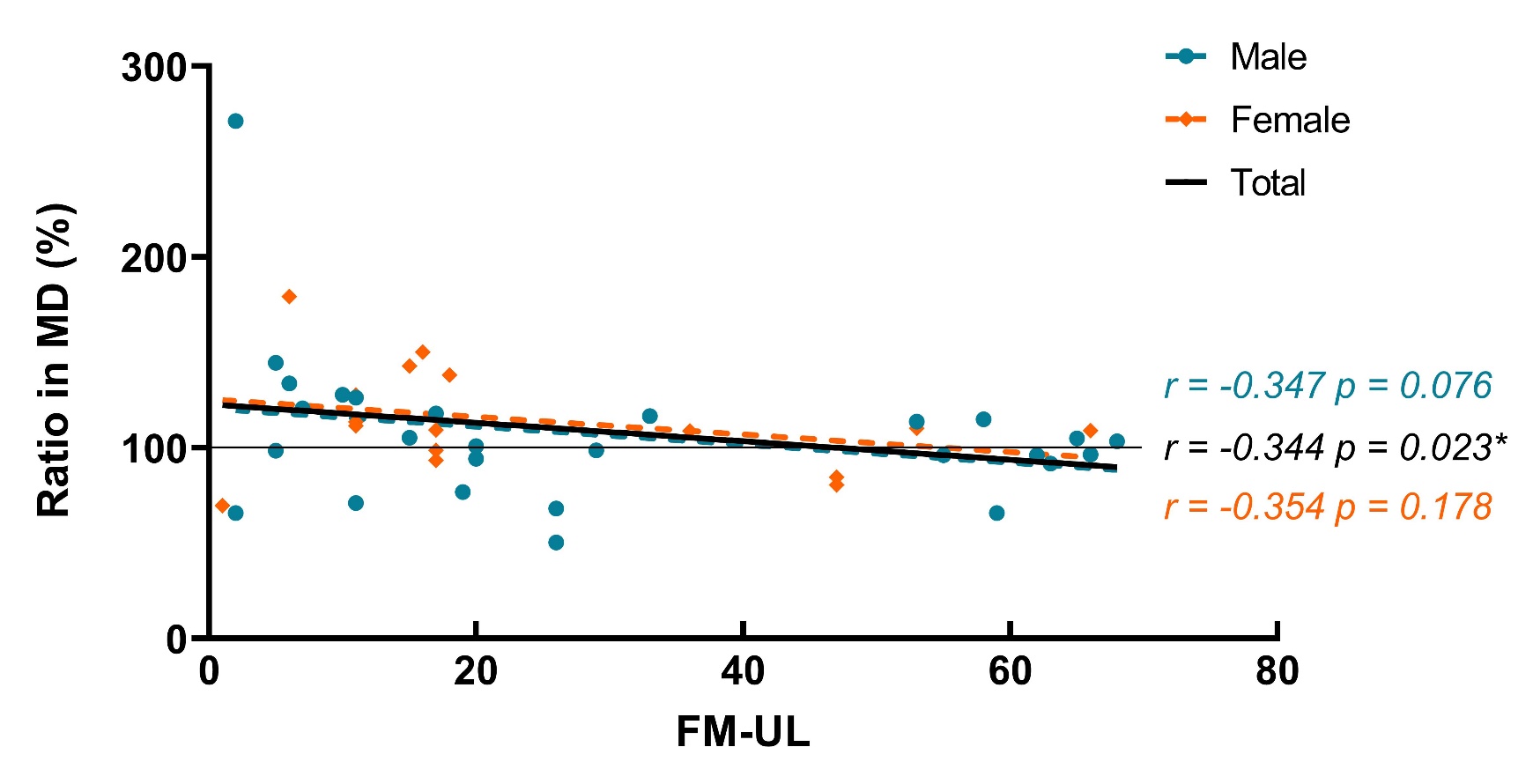


Figure S4 The association between motor function of upper limb in Fugl-Meyer Assessment and PPT ratio in middle deltoid muscle among male, female and total post-stroke elderly, respectively


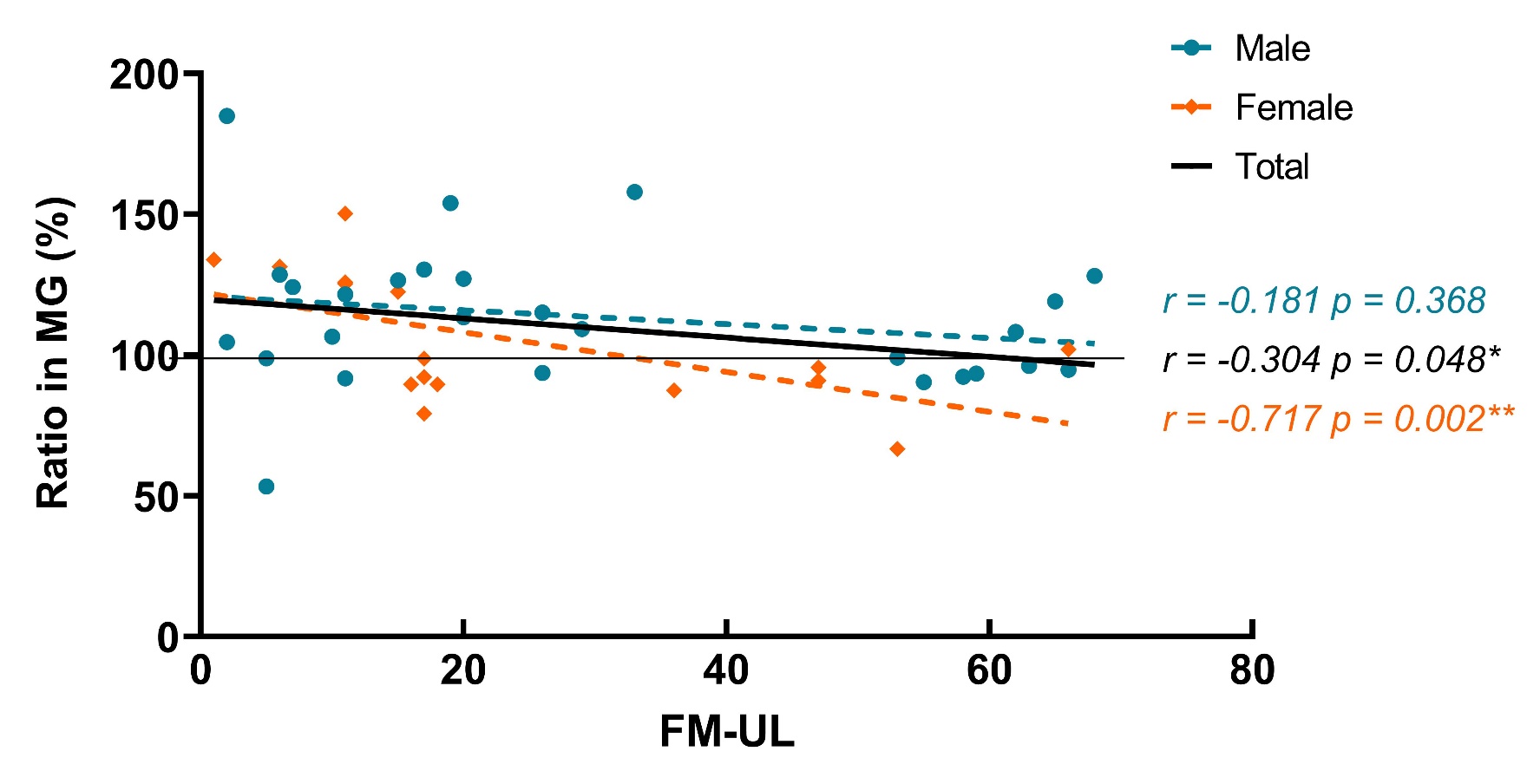


Figure S5 The association between motor function of upper limb in Fugl-Meyer Assessment and PPT ratio in medial gastrocnemius muscle among male, female and total post-stroke elderly, respectively
